# Supplementary material for: Patterns of Intron Gain and Loss in Fungi
Source: PLoS Biol. 2004 Nov 30;2(12):e422. doi: 10.1371/journal.pbio.0020422 (PMC532390; doi:10.1371/journal.pbio.0020422)
Supplement: Table S1 — Also available at http://genes.mit.edu/NielsenEtAl/. (4.3 MB ZIP). [file pbio.0020422.st001.zip › NielsenEtAl/html/753.html]

AN2076.1.NCU04840.1.MG00454.1.FG08491.1


```
 CLUSTAL W (1.82) Multiple Sequence Alignments - Introns Inserted


Sequence 1: NCU04840.1	1028 aa
Sequence 2: MG00454.1	955 aa
Sequence 3: FG08491.1	927 aa
Sequence 4: AN2076.1	974 aa
Alignment Length: 1072 aa
Number Identitical Residues: 224 aa
Alignment Score (without introns) 11993


MG00454.1 	MHQQSRYPPRSSSPANSPQTNPTRTNNSRDAPGTSSRSRASSSAAGPDISGAE--MGDPG
NCU04840.1	MHQQSRHPPRVSSPAVSAQTNPARTNNQRDPRSRTSTDAADRDGAGGGGQDGG--GGALA
FG08491.1 	MHQQPRGPARVSSPGATTQPNLPSRSNSTREAALGSRPRAGSNLVGRDVPNSPSLESPPI
AN2076.1  	MHQQPRSP----APAASTSSARRSQDYDRRSDSPAFDLRTHTNRGLGIETENDTAEPEQS
          	****.* *    :*. :...     . .    .      :  .          :      

MG00454.1 	SGPSRDSVKKLDQIVQ0NFYNKAAVLIIQARLNTTP------------IVAAASGRGTSK
NCU04840.1	HAASRDAIKKLDQIIQ0NFYYKAAVLIIGSRQHLTPGPKKGFNKWVGFHFFKPLPRQAAD
FG08491.1 	PAPPADSVRKLDQIIQ0NFYAKAAVLVLDSRIKSRP---------------ARGANGARK
AN2076.1  	SQPGPEAISKVNQIVT0NYHTKAALIILHSRVELPP--------------SYAKNSNVPR
          	  .  ::: *::**:  *:: ***:::: :* .  *                     .  

MG00454.1 	TNKW0FQIETDEMDDFREDLRIWRTCGSFENRPPPMIIETYLDASCLSPTQSLVVAGDNG
NCU04840.1	DEMQ~FSIETEDTEDFKEELRLYRNSDSFTNRPPPMIIETYIDASRISSSHSLALVDANG
FG08491.1 	PNKW0FQIETDEIDDFRDELKIWKNCGSLDNRPPPMVIEVYLDASRLKDSQSLVIVDENG
AN2076.1  	VNRW0FNVELEETDALKDQLKTWRTCDATDNRPPPMIIETYLDTAGLTNNQTLVALDDNG
          	 :   *.:* :: : ::::*: ::...:  ******:**.*:*:: :. .::*.  . **

MG00454.1 	KMYDVLEALNSSDSASDGSSSSRSRKRNTEVVLERWKIELKYSQNPDDIPDFGPMLPTIY
NCU04840.1	KRWDVLEALNSSESSEDSPRKRHLQKRNTEVILERWRVELKCIPNDDALQDFGPSLPTIY
FG08491.1 	KRWDVMEQLNSYGSSTDSSGASR---RNNEVVIERWQVELKHSGMTS--VDFGPILPTVY
AN2076.1  	KRWDVTESLAASQSSRPAKASSS---RAVDVILERWRVELGDMPGKLP-SDLGAILPTVY
          	* :** * * :  *:  .        *  :*::***::**          *:*. ***:*

MG00454.1 	KRAIVFFRSLFSMLRLVATWKVLQKQPSIARGGHSALQLHCRFVFKES-DTAGVDLLRQP
NCU04840.1	KRSIVFFRSLFLATRVMPGWKFS--QQTMTKGIHPALEVKCRIVPAEA-EFLNYDPLRQP
FG08491.1 	KKAIVFFRSLFITTRLLPAWKFA--SQGAAKNSHPALIPRCRIRLSQP-DRPRYDQLRLP
AN2076.1  	KKSIILFRSLFTYSKFLPAWKFIK-RNGRSR-AHPALRVKYRIFSGHARDLSKQDHLTMP
          	*::*::*****   :.:. **. .     ::  *.**  : *:   .. :    * *  *

MG00454.1 	LSPQHDGRDAVSDYVFGDLEVPVGRFYASVTSRNERNFRVDDTDVTAGSRFMTYEDNLFQ
NCU04840.1	LS---DSKDVVTEYMFGDLEVPVGRFNASVIYRNDCTFRVEDADALLSSRLMGVDEDFFK
FG08491.1 	ID---GRPDPVTEYVFGDLEVPVGRLSTLVTYRSDCNFRVDDSEALLSSRFMGVDENFFR
AN2076.1  	LF--EGDTKVVDTYSFGVTDSPAGPFSVQVTYRTCCDFRVDDSEALLSSQFMGADDEIFQ
          	:    .  . *  * **  : *.* : . *  *.   ***:*::.  .*::*  ::::*:

MG00454.1 	PTLPQR----HSGRRDSFTETGIAIPRRHGKQVDQPEQQTYGSLSTFHGAG-ALGTSPLS
NCU04840.1	PSIPQRPQPVSHGRADSYAEPGSLPSHRLGRRSREP-HQTYGSLSTFHGTG-AFGTSPIS
FG08491.1 	PSLPQQ----HATSRAPAAEAGSLRDHRS-KPNLNDIQQAYGSLSTFHGNV-PIGTSPIS
AN2076.1  	PSLPSG-----GLDARVTPEVGSAPLTRR-TVEDPDLSRAYGSLSTFHHVGPTTGASPIS
          	*::*.             .* *     *          ::********   .. *:**:*

MG00454.1 	ALKAVRPAGSDTDSPTSSAPASVG-PDPPHSLPIAGQSSLSRPSLRGTDGIARRTSVSFQ
NCU04840.1	ALKAVRPAGSDTSSPPGSSAASVDQSEPSHSLPIRSASSR---TLRDVEG-SRRPSISFQ
FG08491.1 	ALRSVRQPGSDTSSPPESIPAQHDVGGPS-SLPVRQGTAR--PHLPALEGLGRRPSVSFQ
AN2076.1  	ALRAAREARASSPSPPTS--SHRNSFAAARASPVGRAATL---ANDTNPNVARRPSISFQ
          	**::.* . :.: **. *  :  .   .. : *:   ::          . .**.*:***

MG00454.1 	PFKAGSLSGSPRLQDLD-LPASPSSLARQS--AALHAARQRNSLTSGMAAS-LRG---GP
NCU04840.1	PFKAGSISGSPRFPDHG-APASPHSLPRPSGLSGISQARNRSSLTAGMAAS-LRGPGAAV
FG08491.1 	PFKAGSLSGSPVPRQLDAEPASPQSLTRPG-IPSLRQAGNRTSLTAGMPAS-LRGGPPTS
AN2076.1  	PFKAPPLSASPSLVDPP-LSASPRTTGAGR--TSLSDSRHMPPPSVTTSSRKPPSFGPDN
          	**** .:*.**   :    .*** :       ..:  : :  . :   .: .  .  .  

MG00454.1 	PIDIAGSASPKPA--SRYSSSFTHRKGKPSYSG-ANRGDDDQGSSGKQSLSSSVQ-PGSG
NCU04840.1	PTDIPKSSSPRPQ--SRYSSSFSHRRSRTSFGGQSKTGDDEASSSGRQSLSSSAQ-PGSA
FG08491.1 	SGETAVAGSPRPASTSRYSSSFTHRRGRLSFGGASKAGDDEQGSSGRQSLASSVAQPGSG
AN2076.1  	ANSSPNSASPRPTPMSRYSSAFSHRRGRPSSGGINKLEDDN--SSGRASATSSGAQPGSG
          	. . . :.**:* . *****:*:**:.: * .*  :  **:  ***: * :**  .***.

MG00454.1 	FLAEAATGTSSGSVQTDDENISEFLKILDSKRTLQSFEP-TSK-TTEAATKRTVAQLSKF
NCU04840.1	LLAEPVGGVASSSSFQTDDDISEFLKELDNRKKLPSFEA-TKKGEQSAAARRTAAQLNKF
FG08491.1 	LLAEVAG-TSSESLRDDNEQLEDFIKALDSKKTLQSFGP-SKT--GESATNKTVAQLSRF
AN2076.1  	LLAEITG-TSSDSIHADDENISEFLKMLDLRKDLLSPSSQTAMDNHSRRMTAASAALSRF
          	:*** .  .:* *    ::::.:*:* ** :: * *  ..:  .  .     : * *.:*

MG00454.1 	HMMRESNNALTESMTS-SINLQPSPSASSRQLSGVP--LLGP----SSMSPSSSP--KPL
NCU04840.1	HLMRDSNNALTESMLMGSSTLHRSSSSSSRHISNMPGSLGNPGISMSGASVSSSPGGKPL
FG08491.1 	HMMRDSNNALTESMTS-SVQMQRSSSSSSRQLTSVPG-MTAP----ASVSASSSPG-KPL
AN2076.1  	RGMKDSNAALSDSMSS-SLLMNRSSATSSKQLSGVPG-IAGT-----SISTASSPGGKAI
          	: *::** **::**   *  :: *.::**::::.:*. :  .     . * :***..*.:

MG00454.1 	SPHTPHTPAVPSRLSEAAIIEHHTQAH----------EIADRVAAQDDHDLGVEASTTRR
NCU04840.1	SPHTPHTPAIPSRLSENSIIDYPAQASYNRRGRSHVVPTEEEEEEEEEREDGDETTQTDR
FG08491.1 	SPHTPHTPAIPSRLSENSIIDYSGQGR----------ITSRQGRTSDNTQPGTIRENTIT
AN2076.1  	SPHTPHTPAIPSRLSSNSIVDNTTTRL-----------HGDNGSPVEEDASDETTTERLP
          	*********:*****. :*::                    .    ::   .        

MG00454.1 	EGTT-------AIDIPLSPRVLQT--ANRRSSSVAHQNRALLDNE--EPVPDLPFAGNRS
NCU04840.1	QQETGGGAVSAAIDIPLSPRLLHAGSANRRASSVAQQHRSLAAGAGGGTGEDDSDIAQRS
FG08491.1 	QDGT------TAIDIPLSPRLATY---QRRASSVAIQNRSMADDD-----DTDSAFAHRS
AN2076.1  	STVT-------AIPIPTSPATIFP-STFRRSSSAANRRSSHAIDD-------DEIFTMRS
          	.  *       ** ** **      :: **:**.* :. :   .              **

MG00454.1 	ISLGADGRE-PTRLTGLGLS---------RGSRSSGNSASLR------SADVRPAGAPRS
NCU04840.1	LSLGAGGGEAPSLSTLLAFQ---------RRNSGDGNEDSSAGSGMAGMQQLQPAADIRA
FG08491.1 	ISLGADDREPPTLSILLGRQ---------MQLEEDSTQRPSDR-----LEPAADTGSTET
AN2076.1  	VSLGAEEPSHTTLGALQRQQDYEPIGTNIAPLESGARSSGGDDGNVLRGPTRRGPGAHRD
          	:****   . .:       .. .. .:.      .. .     ..         ..  . 

MG00454.1 	SSMSTD-PDDKNPSPALISGGASSGSSWKPRFT---------GSLGRGQQQQPPQQRLTP
NCU04840.1	SAELRQ-ANEDQPSNPPVRLMTTSRTPYKSRYSYHGTTTS--STQGRGSGIG-PGARGTP
FG08491.1 	PDMLRQGLSEENPPEGLIPAATSS------------------SPFGRRRYMGMASHKQTP
AN2076.1  	ASLSGPTVATSPYSHHPLHQRRISHSRGRGFSGGPHSLSSGSSSIARGGLIHPYPAEREA
          	.         .  .   :     * :          : ::.:.. .*         .  .

MG00454.1 	PHSS--RGSFSGS---NAGGRYGGSGSRGAG-DSAEADDEPFMFTMSEIERHGQGRTSLD
NCU04840.1	PQSS--GGPFTGM---RYVGTMAGRGGMGVGQGEDGTDDEPLLFAMSELERPS--RHSLE
FG08491.1 	PQSS--RGSFNG------------SLNRQVRGDDDSVNEEPLVFDLSEMDPQG--RRSIE
AN2076.1  	ERDANAGASHSGTEDRRGTGRGSGGGRHNLPQAAQVEEDEPLLFAMSDFGASR---RSFE
          	 :.:.: ....* ..    .  ..             ::**::* :*::        *::

MG00454.1 	EARPG-----------------GEGSGAIESGEKIRGGRYEARGITRRGW----
NCU04840.1	EARGGGVLGGSPRGLRGGAQGAGEASGNTDGGGVGDGEEYEVRGTTRRGW----
FG08491.1 	EARSG-------------------ASGGPNIGP--DRGGYESRNASRRGW----
AN2076.1  	EGRQG-------------------NHGPDSSGNTGSSRRGSGKRGTLSGFHLWP
          	*.* *                     *  . *        . :  :  *:   .
```
